# The Relation of the Profession to the General Public

**Published:** 1901-03

**Authors:** R. R. Kime

**Affiliations:** Atlanta, Ga., Ex-President Tri-State Medical Society Alabama, Georgia and Tennessee; Ex-President Atlanta Society Medicine; Member American Medical, Southern Surgical and Gynecological, Tri-State Medical, State Medical, etc., etc.


ATLANTA

Journal-Record of Medicine.

Successor to Atlanta Medical and Surgical Journal, Established 1855, and Southern Medical Record, Established 1870.

VOL. II. MARCH, 1901. No. 12.

BERNARD WOLFF, M.D., EDITOR, Nos. 319-20 Prudential.

M. B. HUTCHINS, M.D., BUSINESS MANAGER, No. 64 Marietta St.

Published Monthly.

### ORIGINAL COMMUNICATIONS.

### THE RELATION OF THE PROFESSION TO THE GENERAL PUBLIC.*

BY R. R. KIME, M.D., ATLANTA, GA.,

Ex-President Tri-State Medical Society Alabama, Georgia and Tennessee; Ex-President Atlanta Society Medicine; Member American Medical, Southern Surgical and Gynecological, Tri-State Medical, State Medical, etc., etc.

*Mr. President, Members of the Tri-State Medical Society:*

Again I thank you for the honor you have conferred upon me, and assure you I prize it above any professional honor I have ever received.

It has been said a "prophet is not without honor save in his own country," which is usually true; however, forty-three years ago I first saw the light of day on Tennessee soil, and my father received his medical education in Nashville, a Tennessee city.

* Presidential Address Tri-State Medical Society Alabama, Georgia and Tennessee, at Chattanooga, Tenn., October 11 to 13, 1900.

I simply returned to my own native State to receive the honor I prize so highly. With this honor come duties and responsibilities, among which is the custom of an annual address.

This duty I would gladly evade, but I have not the courage to change the custom, hence have selected a subject about which I can write most, express but little, and know less: The Relation of the Medical Profession to the General Public.

The medical profession in its broadest sense has a greater usefulness and more intimate relation to the public than any other profession. In olden times the physician and clergy were one. The humble Nazarene healed both the physical and spiritual.

The true physician of to-day deals not only with the physical, but the mental and moral nature of mankind, and perchance even the spiritual at times. He lives not alone for personal individual success, but upon that broader plane that elevates, develops and improves mankind.

His duty is not only to relieve the sick and suffering, but to uplift the fallen, encourage the weak and warn the erring of the consequences following vice and immorality. It is well for us to pause occasionally and meditate upon our position and relation to the general public, whether it be for good or evil.

What I shall say is not in a spirit of criticism, but with an honest endeavor to stimulate thought and action in a direction that will benefit humanity at large.

As physicians, students of human nature and the physical economy, we are expected to know more of the physical, mental and moral needs of humanity than any other profession. When we consider how closely these three attributes are linked, one interdependent on the other, then we recognize the necessity of every physician studying these higher characteristics of mankind.

Some physicians seem to think they have but little to do with the mental or moral, and have only to deal with the imperfections of the physical. How shall we recognize the abnormal except as a deviation from the normal, and how shall we deal with an effect other than empirically, unless we recognize the cause? Can we have a perfect mental or moral condition without a proper physical basis? Can the physical long remain normal with a diseased mind, or a depraved moral nature? "The evil that men do lives after them, the good is often interred with their bones." So the physical diseases and defects are buried with the body, while more frequently the mental and moral characteristics and defects are transmitted to the third and fourth generations.

We would gladly relieve ourselves of the responsibilities of considering these higher characteristics of mankind as physicians if it were possible, but can we consistently and conscientiously do so and discharge our duty as physicians and benefactors of the human race? I think I can safely assert that at least one fourth or one third of the diseases and ailments the physician is called upon to treat are due to immorality and intemperance, and if considered in a broad sense, fifty per cent. It is evident such diseases are preventable by proper instruction and moral influence. To this influence the physician does and must contribute, else cease to exist. He either helps to build up or tear down by precept, example and advice. He cannot be neutral and make a success. He unconsciously exerts an influence in the community in which he lives for good or evil over his associates and those who know him in proportion as his ability is recognized.

Influence once given out and words once spoken "are as white-winged birds that take their flight never to return again."

In advising patients the words of the physician should carry weight, wisdom and discretion, for they are ofttimes instructions to health and happiness or to suffering and misery. There is no profession whose responsibilities are greater than the one which professes to be able to recognize and repair discord occurring in the "harp of a thousand strings." It has been truthfully said we are fearfully and wonderfully made.

It is even more wonderful how the tissues resist and eradicate disease, repairing the damage done, even at times overcoming the evil influence of treatment given.

Some diseases are necessarily chronic in character and prone to exacerbations, manifesting pain and nervous phenomena.

Especially is this so in neurological and gynecological work. We as physicians too often resort to stimulants and narcotics in such cases. Such remedies frequently give temporary relief, but at too great a sacrifice to the future welfare and interest of the patient.

Our duty is to search for and remove the cause rather than to prescribe a remedy that is liable to produce a condition worse than the disease from which the patient seeks relief. The indiscriminate use of the coal-tar derivatives, cocoa mixtures, coca-cola, stimulants and opiates by the public and profession should be condemned.

The profession, as a body, should use its influence against this growing evil affecting the present generation and try to check to some extent its blighting influence on generations yet to come.

When scientific investigations and experience demonstrate that permanent evil influence often follows a given remedy not alone on the individual taking it but to their offspring, it is a great responsibility to prescribe that remedy.

Dare any conscientious physician pass over such a question lightly? If he is not his brother's keeper, he professes to be his competent counsellor and adviser and morally responsible for the counsel and advice given.

If you had no knowledge of medicine would you ever forgive a physician for prescribing a remedy for your wife or child permanently affecting them physically, mentally and morally, when other remedies equally efficient were at command, and especially when the other remedies are not attended with such evil results?

The prolonged use of opiates and stimulants to pregnant and nursing women is fraught with evil and should be condemned for the sake of countless, helpless victims yet unborn.

At one time it was thought the physiological and therapeutical action of alcohol was well understood, but more recent investigations have cast a cloud over the time-honored customs in its use. Now its food value is a question of doubt; surgical operations, including abdominal sections, are frequently done without its use; typhoid fever and septic conditions in many instances treated as successfully without its use as with it.

Abbot and Lattinan (Jour. Amer. Med. Assoc., Sept. 8, 1900, p. 628), by experiments on animals, find that alcohol increases the susceptibility to infection, and that animals to which alcohol has been given in large or small doses die quicker from infection than those to which it has not been administered.

I am told by a physician who treats many cases of opium habit at his sanitarium, that at least ninety per cent. of the cases contract the habit from physician's prescriptions.

OPIATES IMPORTED INTO THE UNITED STATES.

| | |
|---|---|
| 1871 to 1880 | 3,860,000 lbs. |
| 1881 to 1890 | 4,917,000 lbs. |
| 1891 to 1900 | 6,395,000 lbs. |

and 208,000 ounces of morphia.

And this amount notwithstanding the amount of coal-tar derivatives used of late years for relief of pain.

In view of these increasing evils we should guard well the interests of the general public and those seeking our advice and counsel, in not prescribing a remedy fraught with so much danger unless the indications are definite and positive for its use, and then only when no other remedy less objectionable will answer equally as well.

One man fighting alone can accomplish but little in this day of trusts and organizations of all kinds, but a united profession of three States might accomplish much.

It is proverbial "doctors disagree," yet they are the most sacrificing and noted for adherence to duty when once it is known. Would that I had the power of pen and tougue to express the opportunities of the medical profession for the uplifting of humanity and bettering the condition of the human race as I view it. No profession is better qualified than the medical profession to study the social problems that affect our race.

Being fitted and qualified for that duty carries with it a certain weight and responsibility if that work is left undone. The time is opportune and no better than the present meeting to inaugurate a movement that will bless humanity, lessen disease and suffering. Let us establish a committee in conjunction with the Tri-State Medical Society, representing Alabama, Georgia and Tennessee, to study social questions and problems affecting the development of the human race. A committee of such a character working in conjunction with their respective State Medical Association, uniformity of action can be secured and much good accomplished. Each member of the committee, one from each State, should serve for a term of three years, acting as secretary, treasurer and chairman, one year each respectively; the State committeemen to be selected the same year as president from the respective States. If desired to enlarge the committee, the president and State committeemen may be empowered to select three or five additional members of the profession from their State to serve on the committee. This committee should make a report at each annual meeting of work accomplished in each State.

It is also suggested that the afternoon and evening of the first day of the annual meeting be set apart for the report of this committee, with its recommendations and papers on social questions either by members of the committee or persons selected by them to present papers on such subjects. We cannot at this time enter into a discussion of all the phases of this question nor present it in a way its importance demands. Yet we will endeavor briefly to call attention to a few salient points bearing on this subject.

The physician, knowing more of biology and the relation of mind to matter, is most likely to study social questions from an unbiased standpoint. The study of these questions involves the laws of heredity, the influence of environment, and the value of training. The prevention of crime is a subject of no little importance, and especially so long as criminals and degenerates are allowed to propagate their species *ad libitum*.

In the study of so grave a question sentimentalism should be banished and the cold facts of logic applied, if need be experimentally at first, that we may ultimately reach the truth in matters pertaining to criminology.

The habitual criminal has no right to propagate and should be rendered sterile or not be allowed to marry and bring into the world helpless victims, doomed to destruction even before they are born. Such a course would prevent their reproduction and deter others from criminal acts. The diseased may have a moral right from a personal standpoint to get married and propagate a diseased race, but for the best interests of humanity certain restrictions should be thrown around this class.

The syphilitic, the marked tuberculous, either by acquirement or strong hereditary taint, should not marry; they should forego that pleasure, relinquish their personal rights and privileges for the sake of their would-be offspring and the general good of the public. Here it is the knowledge of the medical profession is essential to protect the public, do justice to the individual, and shield the innocent.

Medical knowledge is essential in the regulation and control of venereal diseases, which are on the increase and will be more so as a result of the Spanish-American and Philippine wars. This question involves moral and physical problems that must be well considered before its proper adjustment can be attained.

